# Natural history footage provides new reef fish biodiversity information for a pristine but rarely visited archipelago

**DOI:** 10.1038/s41598-020-60136-w

**Published:** 2020-02-21

**Authors:** Libby Liggins, Jenny Ann Sweatman, Thomas Trnski, Clinton A. J. Duffy, Tyler D. Eddy, J. David Aguirre

**Affiliations:** 1 School of Natural and Computational Sciences, Massey University, Auckland, New Zealand; 2 Auckland War Memorial Museum, Tāmaki Paenga Hira, Auckland, New Zealand; 3 Department of Conservation, Auckland, New Zealand; 4 Centre for Fisheries Ecosystems Research, Fisheries and Marine Institute, Memorial University of Newfoundland, St. John’s, Canada; 5 School of Biological Sciences, Victoria University of Wellington, Wellington, New Zealand

**Keywords:** Biodiversity, Biogeography

## Abstract

There remain parts of our planet that are seldom visited by humans, let alone scientists. In such locations, crowd-sourced or citizen scientist data can be critical in describing biodiversity and detecting change. Rangitāhua, the Kermadec Islands, are 750 km from the nearest human-habitation. Although our knowledge of this near pristine location has increased with recent biodiversity expeditions, we still lack comprehensive understanding of the marine biodiversity surrounding the islands. In 2015, professional underwater videographers were commissioned to produce a nature documentary focused on Rangitāhua’s reefs. We strategically surveyed the raw documentary video and examined how biodiversity estimates differed from traditional scientific surveys. We uncovered three new fish species records for Rangitāhua, extending the known distribution for each species, two of which are also new records for New Zealand waters. Comparison of documentary video footage with scientific survey methods showed that estimates of reef fish species richness from the documentary video were similar to stationary surveys, but lower than non-stationary surveys. Moreover, all survey methods, including documentary video, captured different fish assemblages, reflecting each method’s particular bias. Overall, we provide a proof-of-concept for how collaborations between scientists and professional natural historians, such as videographers and photographers, can provide valuable biodiversity information.

## Introduction

Despite the efforts of scientists to catalogue the global distribution of biodiversity, we continue to record species in new locations (the “Wallacean shortfall”^1^; reviewed in^2^). New species records change our understanding of individual species ranges and improve our biodiversity knowledge for a given location. Our knowledge of species distributions in the ocean is particularly incomplete^3,4^. Even for many relatively well-researched coastal environments, new species records continue to accumulate. In remote locations, biodiversity inventories are often inadequate due to the logistical and financial challenges of implementing long-term research or monitoring^5^. Biodiversity surveys in such areas are often opportunistic and lack continuity, meaning that scientists and managers wishing to understand the biodiversity values of remote locations have to be resourceful when sourcing data.

Alternative means of biodiversity cataloging are now commonplace as modern biodiversity informatics platforms (such as: iNaturalist, www.inaturalist.org; the Range Extension Database and Mapping project, REDMAP, www.redmap.org.au) are geared toward harnessing the collective knowledge of natural history observers in our society. In order for this citizen-sourced data to be incorporated into biodiversity research, there must be some form of quality control^6^. Verification of citizen-generated biodiversity records typically involves a professional scientist checking photographs and associated sighting metadata for accuracy. The citizen-sourced data may then be confidently used to help to fill gaps in our biodiversity understanding^7^. However, in uninhabited and remote locations where physical access is challenging, or where permission from indigenous landowners and other governing bodies may be required, the potential to attain species records via such citizen scientists may be limited^8^.

Similar to scientists, professional natural historians such as photographers and film makers are often motivated to visit remote locations in order to document new phenomena and biodiversity. Such expeditions may capture valuable ecological and biodiversity information. The visual recording equipment used in the film and photography industries is often far superior to that used by scientists and produces images of sufficient quality for taxonomists to identify organisms to species (e.g.^9^). Furthermore, as natural history film makers and photographers access diverse funding streams, they are not constrained by the same scientific and conservation funding rounds. There are several examples where natural history footage or photographs have contributed to a valuable scientific understanding of the marine environment (e.g.^10,11^), but to date these records have largely been *posthoc*, and not coordinated with the source of the photos or footage. Hence, valuable biodiversity records likely remain hidden from our collective understanding.

Rangitāhua (Kermadec Islands) is a chain of small, uninhabited islands ~750 km northeast of New Zealand, spanning 2 degrees of latitude. The shallow reef biodiversity of the islands is a unique mixture of tropical, subtropical and temperate organisms, including 199 shallow reef fishes^12,13^. Although the Territorial Sea (within 12 nautical miles of land) around Rangitāhua was designated as a large no-take Marine Reserve in 1990^14^, there has been no comprehensive survey or formal monitoring of the reef biodiversity. Several recent biodiscovery expeditions to Rangitāhua have described new species records^15–17^; however many parts of the islands and several habitats remain unexplored by scientists^18^.

In this study, we present new species records and biodiversity knowledge for the shallow reef fish assemblage of Rangitāhua (Kermadec Islands Marine Reserve) obtained from raw documentary video taken by professional underwater videographers. In 2015, four underwater videographers undertook a three week filming expedition to Rangitāhua for the documentary series “Our Big Blue Backyard” produced by Natural History New Zealand (NHNZ). The videographers had a brief for the types of shots they were to take, and the ‘characters’ (i.e. feature species) that would form the narrative of the episode. Following the expedition, NHNZ produced a 44 minute episode focused on Rangitāhua, which has been broadcast in many international territories, as well as New Zealand. In collaboration with NHNZ, we studied the raw documentary video to investigate whether such documentary film expeditions can deliver additional biodiversity records for reef fishes, and how the estimates of reef fish biodiversity from such footage may differ from previous, more traditional, scientific surveys.

## Methods

### Description of raw footage and biodiversity data collection

Videos were filmed using a RED Dragon, Panasonic GH4 with SHOGUN attachment, and GoPro Hero 4 between 20^th^ October and 8^th^ November 2015. Videos ranged from 1:33 minutes to 78:04 minutes in duration and each video contained between 1 and 137 different shots (segments of continuous footage). For our purposes ‘shots’ were treated as sampling units. Shots varied in length (0:01 to 13:48 minutes), field of view, spatial area featured, and movement of the camera. For example, in some shots the videographer followed an individual fish whereas in others the shots were taken from a stationary, remote camera. From the raw video available (106 videos and 3439 shots, total) we randomly sampled 22 videos and 1074 shots.

Each sampled shot was classified into one of six categories according to the focal subject or utility for observing reef fishes (see Supplementary Fig. S1). ‘Reef’ was the most common shot type and consisted of reasonably clear imagery featuring reef. ‘Extreme close-up’ was a close-up of, for example, a coral that inhibited the view of any surrounding fishes. ‘Pelagic’ included shots with no visible reef, but excluded any shots that featured whales (classified separately as ‘whale’). Additionally, there was a category for shots taken at ‘night’, another for ‘cinematic’ shots not focused on biodiversity or habitat, and the last category was for shots with insufficient lighting to identify fishes accurately (‘insufficient light’). Shots that were not underwater or were completely black were ignored.

For each shot, the fish species present were identified using reference books^19–22^. VLC Media Player v 3.0.6^23^ was used to view the videos and to adjust brightness and contrast to aid fish identification. Where species identification was not possible, the genus or family identity was recorded. The full list of fish species observed, across all types of footage, was compared to published checklists of Kermadec Islands coastal fishes^13,15^. If absent from these comprehensive checklists, the species was considered a new record for Rangitāhua.

### Comparison of documentary video biodiversity information with other methods of fish biodiversity assessment

To investigate potential biases introduced by documentary video estimates of reef fish biodiversity, we compared the documentary video dataset to three other underwater visual surveys of reef fish biodiversity at Rangitāhua. The documentary video dataset considered only ‘reef’ shots, so that it was comparable to the other surveys of reef fish biodiversity.

Each survey method was different, and each had different objectives. The first was a timed, stationary survey (hereafter ‘timed stationary’) intended to observe both benthic and pelagic fishes, conducted at three locations around the northernmost island of Rangitāhua, Raoul Island: two locations in the south of Denham Bay; and one on the western side of the Meyer Islets (for full study details see^24^). There were 5 sites chosen for each location, and 10 to 13 stations each separated by 20 m at each site. At each station, all the fishes from the sea floor to the surface were identified and counted in a 5 ×5 m^2^ area for 3 minutes.

The second dataset was a timed belt transect survey (hereafter ‘timed transect’) of benthic and pelagic fishes from various locations around Raoul Island and Macauley Island (T. Trnski, unpublished data from October–November 2015). There were 12 locations in total, each with a deep (18.3–24.0 m) and shallow (6.0–11.9 m) transect (the survey depth being dependant on reef topography and sea conditions). At each station, conspicuous benthic and pelagic fishes were identified and counted while laying a straight-line, belt transect (25 × 5 m), and cryptic benthic fishes were recorded on return along the same transect. Each return transect varied in duration between 6 and 31 minutes.

The third dataset was a timed swim survey (hereafter ‘timed swim’) of large predatory fishes at Raoul Island, Macauley Island, Curtis Island, and Cheeseman Island (C.A.J. Duffy, unpublished data from 12–24 May 2011). There were 12 locations each with 1 or 2 transects. Each transect, comprised a straight line timed swim of 20 minutes where large predatory and bentho-pelagic fishes were recorded to the edge of visibility. Smaller benthic fishes were only counted within 5 m either side of the transect. Juvenile wrasses, small pelagic and planktivorous fishes were excluded.

Only survey data from Raoul Island were compared across studies. The species names used across all datasets were standardised to the senior synonym according to World Register of Marine Species^25^ and New Zealand fishes resources^22,26^. All species of the *Kyphosus* genus were lumped as differentiation of the species was inconsistent through time due to confusion about valid species names^27^. Presence-absence data only was used due to differences in the way abundance was quantified (e.g. counts or binned counts) across the different sources (see Supplementary Dataset S5).

### Statistical analysis of biodiversity data

To examine if fish species richness data sourced from documentary video differed from species richness data captured in more traditional, underwater visual census of fish biodiversity at Raoul Island we used ANCOVA implemented in the R Statistical Environment v3.5.1^28^. The response in our ANCOVA model was the species richness of each sampling unit, and data source was a categorical predictor with four levels: documentary video, timed stationary, timed swim and timed transect. The duration of each survey varied widely, and not surprisingly surveys of longer duration encountered more species (see Supplementary Fig. S2); accordingly, time (*log* transformed) was included as a covariate to account for differences in the duration of each survey. The documentary video was set as the reference category in our analysis and differences among levels were explored using treatment contrasts.

To explore differences in fish species composition among data sources we used PERMANOVA + ^29,30^ for PRIMER-E.v7^31^. Similar to the univariate analysis above, we explored differences in community composition (expressed as Jaccard’s dissimilarity) among data sources including time (*log* transformed) as a covariate. After establishing that fish species composition differed among data sources, we explored the distinctiveness and consistency in the species composition observed by each data source using canonical analysis of principal coordinates.

## Results and Discussion

**Figure 1:**
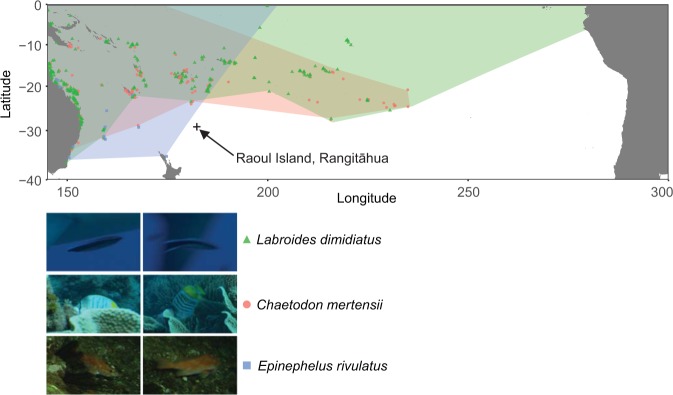
New species records for Rangitāhua. Species range maps^36^ and images (^©^NHNZ) of the new species records for Rangitāhua taken from the raw video footage. Raoul Island, Rangitāhua, is denoted by the cross and is shown to indicate the magnitude of the change in the known ranges for each species.

Review of the raw documentary video footage from the 2015 expedition generated three new fish species records for Rangitāhua: *Labroides dimidiatus*, *Chaetodon mertensii* and *Ephinephalus rivulatus* (Fig. 1). These species are considered tropical (or subtropical in the case of *E. rivulatus*^21^), consistent with previous studies that suggest a characteristic warm-water component in the fish fauna of Rangitāhua^15,32–35^. The observations of the cleaner wrasse, *L. dimidiatus*, and the yellow-back butterflyfish, *C. mertensii* from Rangitāhua represent the first records of these coral reef species for New Zealand waters. Both are cosmopolitan tropical Indo-Pacific species with their closest populations to Rangitāhua occurring at Teleki Tonga (South Minerva Reef) about 900 km to the northeast. *Chaetodon mertensii* also occurs at Norfolk Island about 1,300 km west of Rangitāhua^36^. Thus, our new records are extensions to the known geographic distributions for these fishes. The half-moon grouper, *E. rivulatus* is widespread throughout hard and soft bottom habitats of the Indo-West Pacific with its eastern distributional limit reported to be northeast North Island, New Zealand, Norfolk Island, New Caledonia and southern Japan^22^. This record of *E. rivulatus* from Rangitāhua extends its range eastward by about 700 km. All new records were of mature individuals, making it unlikely that these species represent new arrivals; instead, it is more likely that they were overlooked due to their rarity or residence in unsurveyed sites.

**Figure 2:**
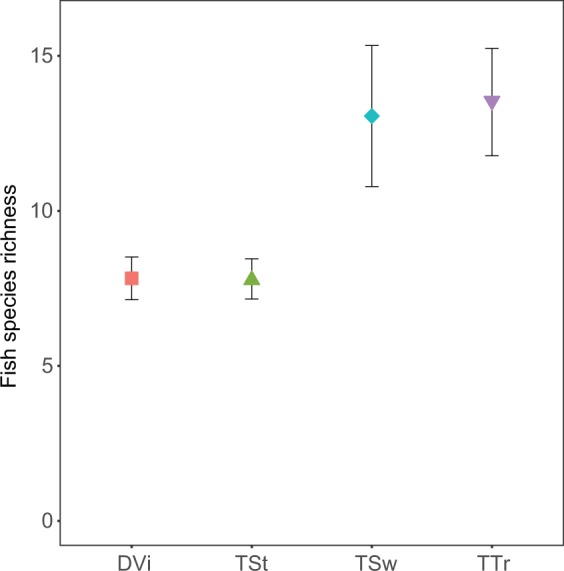
Surveyed fish species richness of Raoul Island, Rangitāhua for the average survey duration of 04:28 (mm:ss). Mean (±SE) species richness of fishes captured by the four different survey methods considered in our study (DVi = Documentary Video, TSt = Timed Stationary, TSw = Timed Swim and TTr = Timed Transect).

The documentary video sampled the fish assemblages for the shortest time period, and accordingly, captured the fewest number of species (see Supplementary Fig. S3). However, after accounting for variation in the duration of each survey, species richness captured in the documentary video dataset was lower than that observed by the timed transect and timed swim (*t*_195_ = 5.683, *P* < 0.001 and *t*_195_ = 5.230, *P* < 0.001 respectively; Fig. 2) but comparable to that observed by the timed stationary method (*t*_195_ = 0.019, *P* = 0.969, Fig. 2). Most of the reef footage in the documentary video we examined was stationary, thus it is likely that that the lower species richness of the timed stationary dataset and the documentary video dataset, was due to sampling a smaller area of reef and/or sampling a lower diversity of habitats than the swimming surveys of the timed swim and timed transect. Such stationary surveys are known to record lower fish species diversity, particularly for sedentary fish species^37^. Furthermore, even in the case of moving reef footage, estimates of species richness from video footage are typically lower than in visual census, due to field of view and difficulty in identifying fishes based on video footage alone^38^.

**Figure 3:**
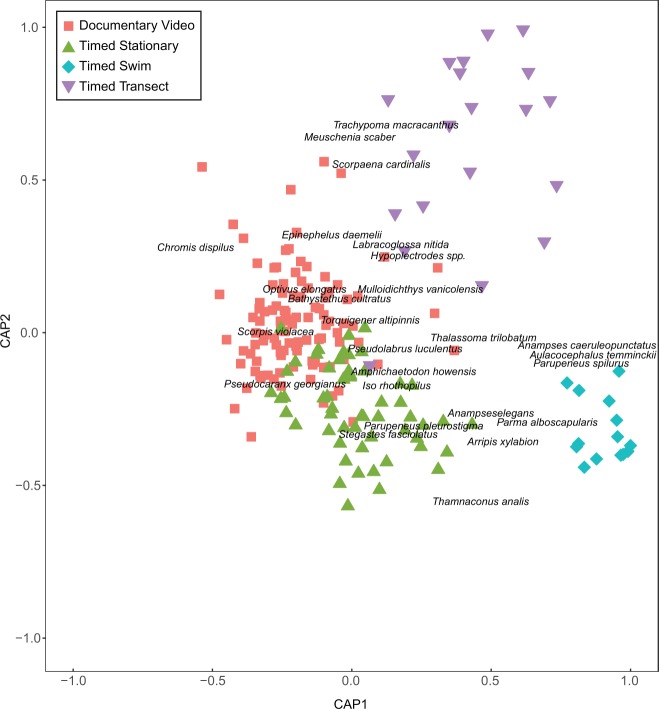
Surveyed fish community composition of Raoul Island, Rangitāhua. Shown are the first and second axes of the canonical analysis of principal coordinates for differences (expressed as Jaccards dissimilarity among samples) in fish community composition at Raoul Island, Rangitāhua for different survey methods. The ten most strongly correlated species (both positive and negative) for each axis are displayed to aid interpretation. Axes and sample coordinates have been scaled to unit length so that they are on the same scale as the correlations.

There were significant differences in the species composition observed among the surveys (*Pseudo-F*_3,195_ = 7.0475, *P*_perm_ < 0.001) and according to the time period surveyed (*Pseudo-F*_1,195_ = 1.9964, *P*_perm_ = 0.009). CAP analyses revealed that the species composition of the timed swim dataset was the most consistently distinct with the highest classification success (100%), followed by the documentary video dataset and the timed stationary dataset (89% and 90% respectively) and last the timed transect dataset (87%, Fig. 3). The high classification success of the timed swim dataset is not surprising as the survey was purposely designed to exclude common bentho-pelagic species such as two-spot demoiselles (*Chromis dispilus*), Kermadec demoiselles (*Chrysiptera rapanui*) and blue maomao (*Scorpis violacea*). These bentho-pelagic species contributed strongly to the first axis of the CAP analysis, which separated the timed swim dataset at one extreme from the timed stationary dataset and documentary video dataset at the other extreme. A second *a priori* bias was the probable focus on charismatic ‘characters’ in the documentary video. Accordingly, fishes such as spotted black grouper (*Ephinephelus daemelii*) and bluefish (*Girella cyanea*) helped distinguish the documentary video dataset from the timed swim dataset. Nevertheless, even when the charismatic characters featured in the documentary video were removed from the analysis, the classification success of each survey method remained very high (> 80%, see Supplementary Fig. S4).

The timed transect, perhaps the most comprehensive and representative of the datasets, was separated from the other datasets on the second CAP axis, also suggesting a methodological difference. The species contributing strongly to the extremes of this axis were cryptic, stationary reef-dwelling species (such as Cook’s scorpionfish, *Scorpaena cardinalis*, and toadstool grouper, *Trachypoma macracanthas*) recorded in the timed transect and species potentially associated with the reef-sand interface recorded in the timed swim dataset (such as the darkvent leatherjacket, *Thamnaconus analis* and the blue-spotted wrasse, *Anampses caeruleopunctatus*) at the other extreme. These differences between the timed swim and timed transect are presumably driven by the survey design (as described in^39^). Specifically, the ‘timed transect’ was a belt transect, where the intention is to survey all fishes within a given area including cryptic fishes, whereas for the timed swim fishes were recorded to the edge of sight, increasing the potential for recording highly mobile fishes.

The disparate objectives of natural history film makers and research scientists will inevitably lead to shortcomings when re-purposing raw documentary video to infer biodiversity information. For example, the documentary video footage was not specifically created with the intention of taxonomically classifying all fish species, therefore not all fish could be identified. Furthermore, although rich metadata was provided in the shot log by the videographers (regarding subject matter, species, data, camera, videographer) this did not include geographic coordinates for the location of filming. Some location information was occasionally provided, and more specific details required cross-referencing the field logs and personal dive logs of the videographers. However, recording geographical coordinates in the shot log for future expeditions should be straightforward. Cameras and diving computers increasingly enable users to geo-reference events, and retrospectively retrieving coordinates using maritime charts or Google Earth is standard practice for many divers^40^.

Our survey of raw video taken for the purposes of creating a natural history documentary has provided valuable new biodiversity information for the remote, rarely surveyed, shallow reefs of Rangitāhua. Our study provides a proof-of-concept that raw documentary video has significant scientific value, and we demonstrate a method for strategically sampling raw footage to provide biodiversity information. Although the documentary video dataset found lower species richness than non-stationary methods and was biased toward large and charismatic fish species, traditional survey methods also recovered different species compositions from one another, depending on the survey design and objectives of each scientist. Overall, our study signals the untapped scientific value of thousands of hours of natural history video that exists throughout the world, which could be used to shine new light on rarely visited locations and retrospectively observe past baselines. There are often commercial restrictions on access to footage and publication of the data obtained from these sources that will need to be reconciled. Nonetheless, we anticipate that scientific inference from natural history video could increase in future as recording equipment able to capture high-resolution imagery becomes more accessible^6^ and advances in computer vision and machine-learning enables automated species recognition^41,42^.

## Supplementary information

Additional analyses.

Dataset S5.

## Data Availability

New species records for Rangitāhua have been added to the forthcoming version of the “Checklist of the coastal fishes of Lord Howe, Norfolk and Kermadec Islands, southwest Pacific Ocean” (Francis, 2019) and the “Annotated check list of the marine flora and fauna of the Kermadec Islands Marine Reserve and northern Kermadec Ridge, New Zealand” compiled by C. A. J. Duffy, with contributions from S. Ahyong (Decapoda) and D. Gordon (Bryozoa)(Department of Conservation file DOCDM-71864). Species presence-absence data for the four survey datasets are available in Supplementary Dataset S5.
