# Supplementary material for: Natural history footage provides new reef fish biodiversity information for a pristine but rarely visited archipelago
Source: Sci Rep. 2020 Feb 21;10:3159. doi: 10.1038/s41598-020-60136-w (PMC7035361; doi:10.1038/s41598-020-60136-w)

Supplementary Information

Title: Natural history footage provides new reef fish biodiversity information for a pristine but rarely visited archipelago

Author list: Libby Liggins, Jenny Ann Sweatman, Thomas Trnski, Clinton Duffy, Tyler D. Eddy, J. David Aguirre

Supplementary Figure S1. Example frames for different categories of footage taken from documentary video (©NHNZ).

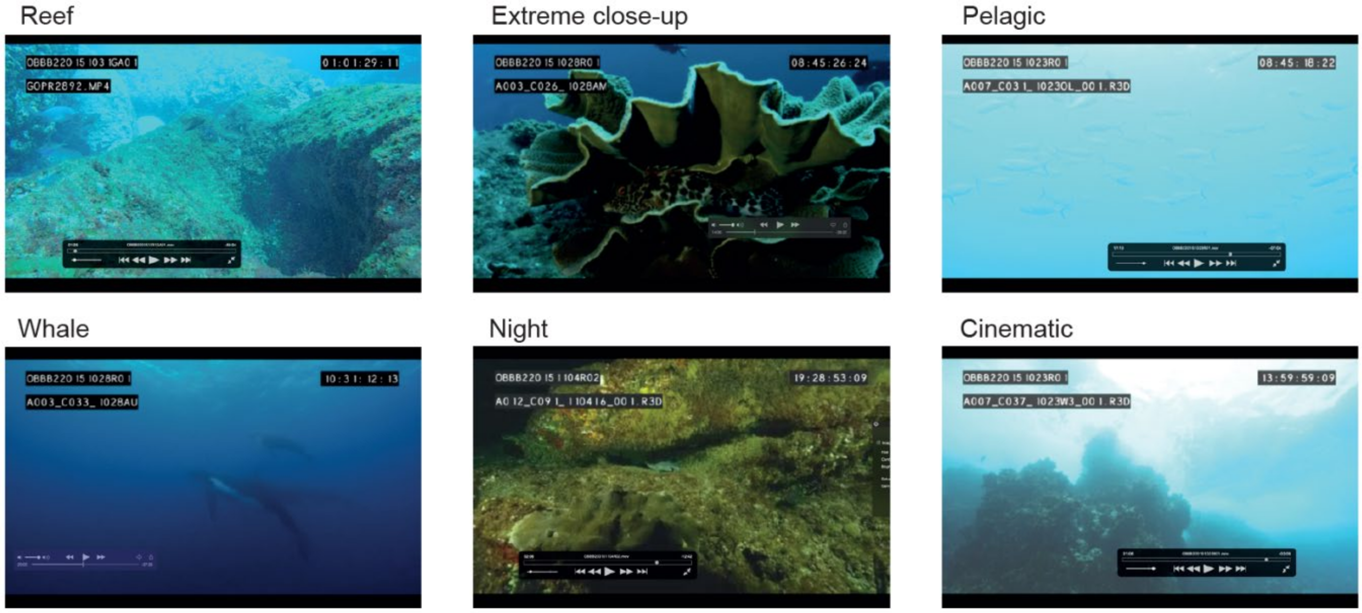

Supplementary Figure S2. Shallow reef fish species richness at Raoul Island, Rangitāhua as a function of time for four different survey methods. The Timed Swim and Timed Stationary methods surveyed for a fixed period of time whereas the Documentary Video and Timed Transect survey methods varied in duration. The plot shows a clear increase in the species richness sampled as a function of time both within survey method (Documentary Video and Timed Transect) and among survey methods.

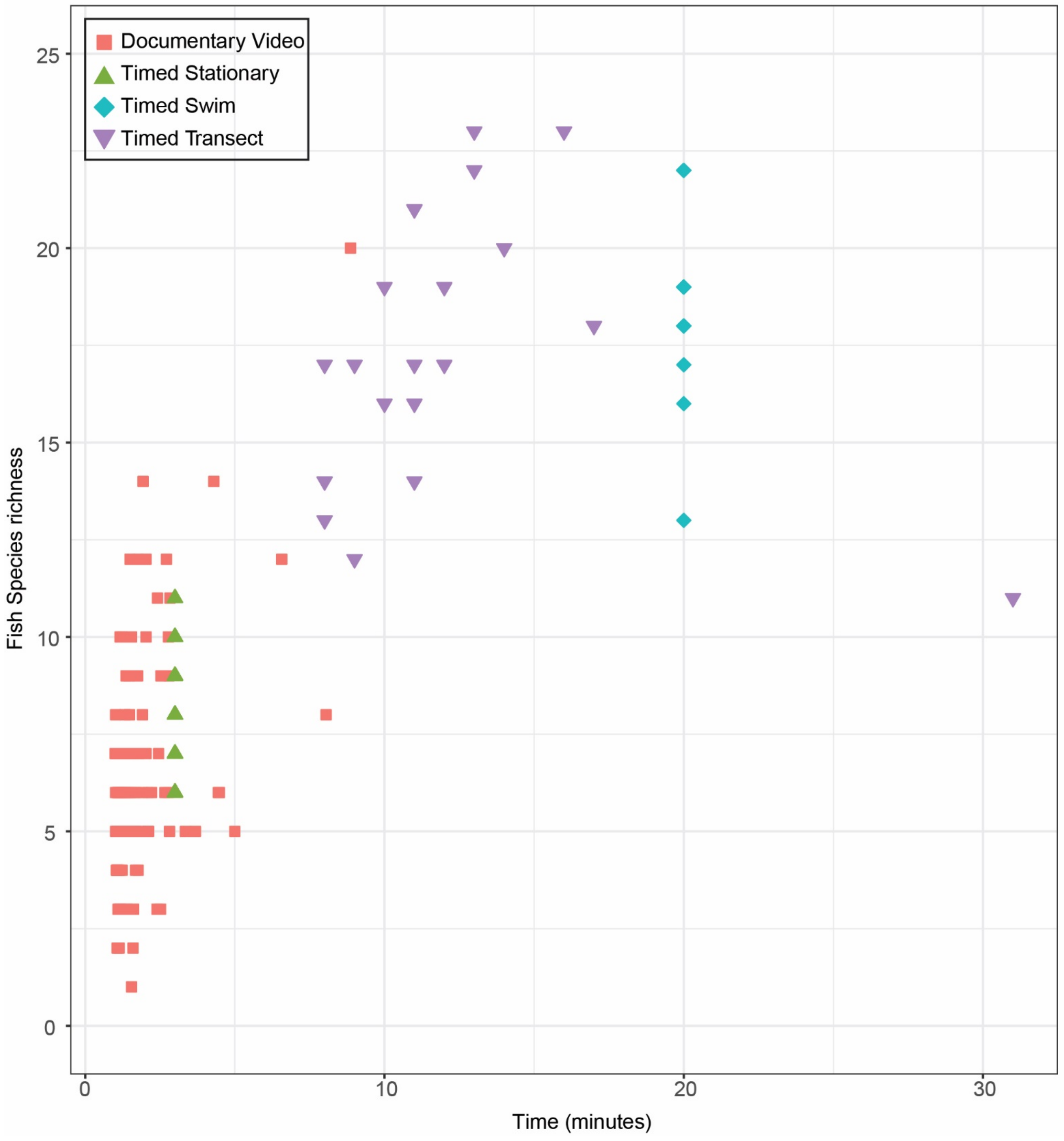

Supplementary Figure S3. Surveyed fish species richness of Raoul Island, Rangitāhua ignoring differences in survey duration (Supplementary Figure S2). Mean (±SE) species richness of fishes captured by the four different survey methods considered in our study (DVi = Documentary Video, TSt = Timed Stationary, TSw = Timed Swim, and TTr = Timed Transect). ANOVA analyses indicated that the species richness captured in the Documentary Video dataset was lower than all other survey methods (t_{196} = 3.966, P < 0.001, t_{196} = 12.265, P < 0.001 and t_{196} = 11.193, < 0.001 for the Timed Stationary, Timed Swim, and Timed Transect, respectively).

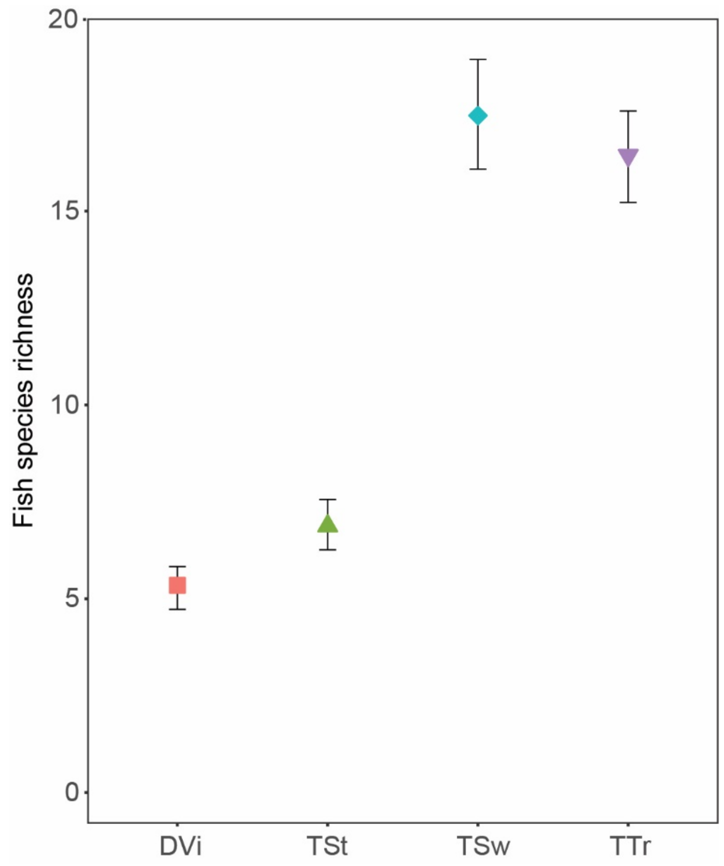

Supplementary Figure S4. Surveyed fish community composition of Raoul Island, Rangitāhua omitting charismatic characters featured in the documentary video. The charismatic fish characters featured in the documentary video included *Chromis dispilus*, *Girella cyanea*, *Scorpis violacea*, *Parma kermadecensis*, *Epinephelus daemelii*, *Scorpaena cardinalis*, *Pseudocaranx georgianus,* and *Pterois volitans*. Shown are the first and second axes of the canonical analysis of principal coordinates for differences (expressed as Jaccards dissimilarity among samples) in fish community composition at Raoul Island, Rangitāhua for different survey methods. The ten most strongly correlated species (both positive and negative) for each axis are displayed to aid interpretation. Axes and sample coordinates have been scaled to unit length so that they are on the same scale as the correlations. CAP analyses ignoring the featured species revealed that the species composition of the Timed Swim dataset remained the most consistently distinct with a classification success of 100%, followed by the Documentary Video dataset and the Timed Stationary dataset (89% and 87% respectively) and last the Timed Transect dataset (80%).

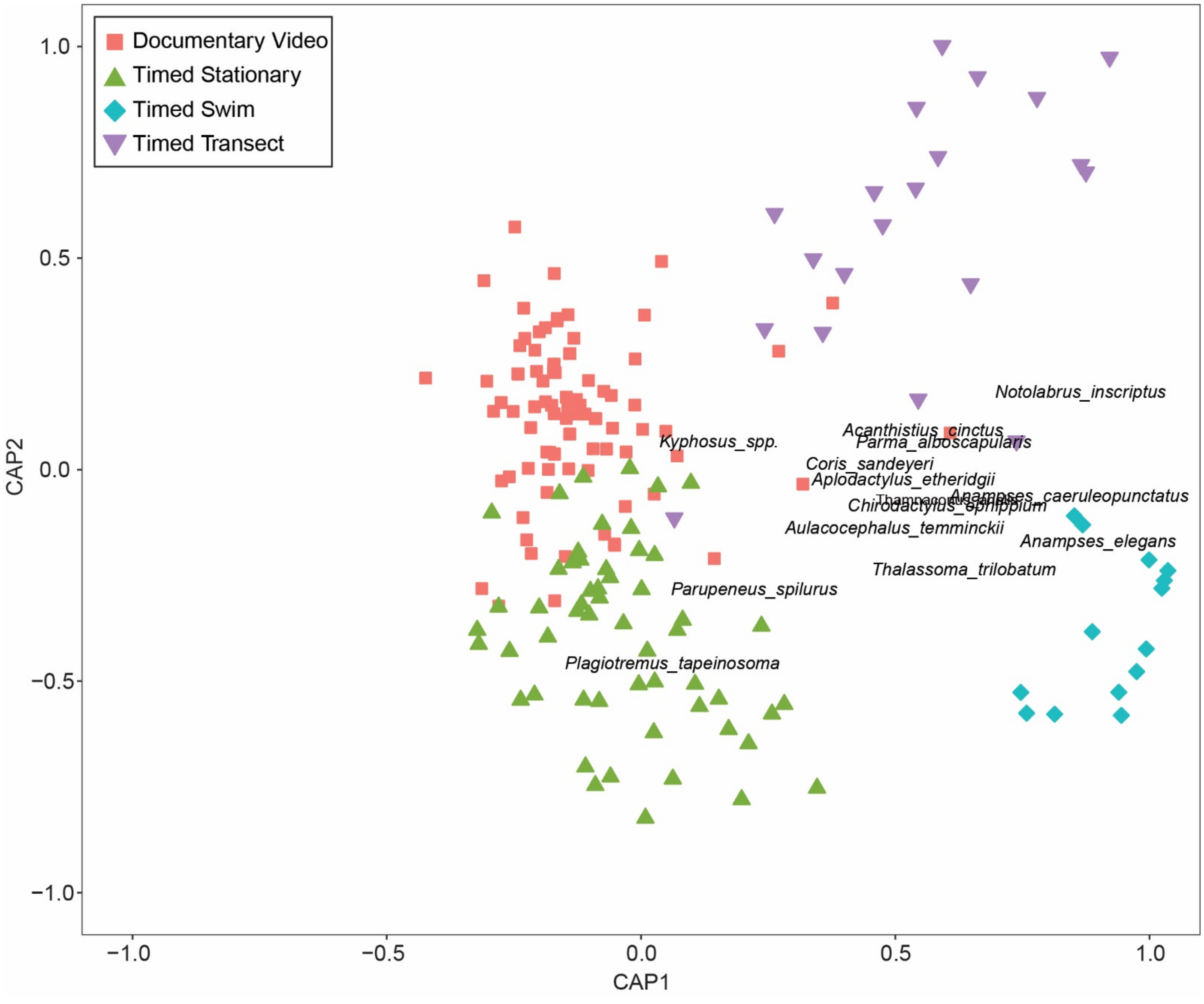

Supplement: Supplementary file 1 — Additional analyses. [file 41598_2020_60136_MOESM1_ESM.pdf]
